# Nox3-Derived Superoxide in Cochleae Induces Sensorineural Hearing Loss

**DOI:** 10.1523/JNEUROSCI.2672-20.2021

**Published:** 2021-05-26

**Authors:** Hiroaki Mohri, Yuzuru Ninoyu, Hirofumi Sakaguchi, Shigeru Hirano, Naoaki Saito, Takehiko Ueyama

**Affiliations:** ^1^Laboratory of Molecular Pharmacology, Biosignal Research Center, Kobe University, Kobe, 657-8501, Japan; ^2^Department of Otolaryngology-Head and Neck Surgery, Kyoto Prefectural University of Medicine, Kyoto, 602-8566, Japan

**Keywords:** age-related hearing loss, drug-induced hearing loss, hearing loss, NADPH oxidase, noise-induced hearing loss, reactive oxygen species

## Abstract

Reactive oxygen species (ROS) produced by NADPH oxidases (Nox) contribute to the development of different types of sensorineural hearing loss (SNHL), a common impairment in humans with no established treatment. Although the essential role of Nox3 in otoconia biosynthesis and its possible involvement in hearing have been reported in rodents, immunohistological methods targeted at detecting Nox3 expression in inner ear cells reveal ambiguous results. Therefore, the mechanism underlying Nox3-dependent SNHL remains unclear and warrants further investigation. We generated *Nox3-Cre* knock-in mice, in which Nox3 was replaced with *Cre recombinase* (*Cre*). Using *Nox3-Cre;tdTomato* mice of either sex, in which tdTomato is expressed under the control of the *Nox3* promoter, we determined Nox3-expressing regions and cell types in the inner ear. Nox3-expressing cells in the cochlea included various types of supporting cells, outer hair cells, inner hair cells, and spiral ganglion neurons. Nox3 expression increased with cisplatin, age, and noise insults. Moreover, increased Nox3 expression in supporting cells and outer hair cells, especially at the basal turn of the cochlea, played essential roles in ROS-related SNHL. The extent of Nox3 involvement in SNHL follows the following order: cisplatin-induced hearing loss > age-related hearing loss > noise-induced hearing loss. Here, on the basis of *Nox3-Cre;tdTomato*, which can be used as a reporter system (*Nox3-Cre*^+/−^*;tdTomato*^+/+^ and *Nox3-Cre*^+/+^*;tdTomato*^+/+^), and *Nox3*-KO (*Nox3-Cre*^+/+^*;tdTomato*^+/+^) mice, we demonstrate that Nox3 inhibition in the cochlea is a promising strategy for ROS-related SNHL, such as cisplatin-induced HL, age-related HL, and noise-induced HL.

**SIGNIFICANCE STATEMENT** We found Nox3-expressing regions and cell types in the inner ear, especially in the cochlea, using *Nox3-Cre;tdTomato* mice, a reporter system generated in this study. Nox3 expression increased with cisplatin, age, and noise insults in specific cell types in the cochlea and resulted in the loss (apoptosis) of outer hair cells. Thus, Nox3 might serve as a molecular target for the development of therapeutics for sensorineural hearing loss, particularly cisplatin-induced, age-related, and noise-induced hearing loss.

## Introduction

Hearing loss (HL) is one of the most common sensory impairments in humans (Muller and Barr-Gillespie, 2015). The nerve pathways from the organ of Corti (OC) in the cochlea to the auditory area in the brain are compromised in sensorineural HL (SNHL). SNHLs, such as age-related HL (ARHL), drug-induced HL (DIHL), including cisplatin (*cis*-diamminedichloridoplatinum II [CDDP])-induced HL (CIHL), and noise-induced HL (NIHL), are classified based on the underlying mechanism (Kamogashira et al., 2015). However, treatment options mostly rely on medical instruments, with no reliable pharmacological interventions (Muller and Barr-Gillespie, 2015).

The OC contains two types of mechanosensitive sensory cells: inner hair cells (IHCs) and outer hair cells (OHCs). The OHCs are surrounded by “supporting cells” (SCs), which include inner phalangeal cells (IPhCs) and outer phalangeal cells (also referred to as Deiters' cells [DCs]), inner and outer pillar cells (IPCs and OPCs), Hensen's cells (HeCs), and Claudius' cells (CCs) (Frolenkov et al., 2004). Hair cells (HCs) at the basal turn detect high-frequency sounds, whereas those at the apical turn detect low-frequency sounds (Schwander et al., 2010). HCs are fundamentally not regenerated after birth in mammals, and OHCs are more susceptible to various insults than IHCs (Atkinson et al., 2015; Furness, 2015). In humans and mice, a typical pattern of HC loss during aging, ototoxicity, and acoustic trauma starts with dominant OHC loss accompanied by minor IHC loss at a later stage in a basal-to-apical progression manner (Kujawa and Liberman, 2019).

Reactive oxygen species (ROS) induce oxidative stress and trigger SNHL (Wong and Ryan, 2015). NADPH oxidases (Nox) are one of the main sources of ROS (Leto et al., 2009) and are the only enzyme family to have ROS production as their primary function (Altenhofer et al., 2012). There are seven isoforms in humans (NOX1-5, DUOX1-2), and previous studies have demonstrated that ROS contribute to ARHL, DIHL, and NIHL (Kamogashira et al., 2015). Recently, we reported a transgenic mouse line that expresses *NOX4*, which produces low levels of ROS without stimulation, exhibits hearing vulnerability after noise exposure (NE) (Morioka et al., 2018), further indicating that ROS contribute to SNHL.

Initially, Nox3 was reported to be essential for otoconia biosynthesis, and mice with functionally deficient Nox3 manifest a “head-tilt” phenotype (Paffenholz et al., 2004). Subsequently, *Nox3* was reported to be specifically expressed at low levels in the OC and spiral ganglion neurons (SGNs) using RT-PCR; however, ISH revealed *Nox3* expression in SGNs and HCs in vestibules (Banfi et al., 2004). Noxo1 is an essential Nox3 activator (Ueyama et al., 2006) whose early truncation results in an otoconia defect (Kiss et al., 2006). It is reportedly expressed at low levels in both sensory and nonsensory epithelia in vestibules and SGNs (Kiss et al., 2006). More recently, expression of p22*^phox^*, an essential heterodimer component of Nox3 (Ueyama et al., 2006) whose deficiency in mice results in an otoconia defect, was observed in the apical surface of epithelia facing the lumen of the endolymphatic sac (ES) and endolymphatic duct (ED) (Nakano et al., 2008). Additionally, *Nox3* mRNA expression in cochleae increased after CDDP administration, and the transtympanic administration of a *Nox3* small interfering RNA (siRNA) resulted in reduced CIHL and apoptosis of OHCs and SGNs (Mukherjea et al., 2010). Conversely, Nox3 was proposed to offer protection against NIHL, especially at a low frequency (8 kHz) (Lavinsky et al., 2015); thus, Nox3 functions in SNHL remain controversial. Although Nox3-expressing cells can serve as prognostic markers for hearing impairment, Nox3 detection remains cumbersome because of a lack of reliable detection methods, barring the ISH-based RNAscope (Rousset et al., 2020). Here, we generated *Nox3-Cre;tdTomato* mice, in which tdTomato fluorescence is regulated by the *Nox3* promoter-driven Cre recombinase (Cre), to investigate cell types expressing Nox3 in inner ears. Furthermore, we examined the mechanism by which Nox3-expressing cells in cochleae contribute to SNHL using *Nox3-Cre*^+/−^*;tdTomato*^+/+^ and *Nox3-Cre*^+/+^*;tdTomato*^+/+^ (*Nox3*-KO) mice.

## Materials and Methods

### 

#### 

##### Animals

**Figure 1.**
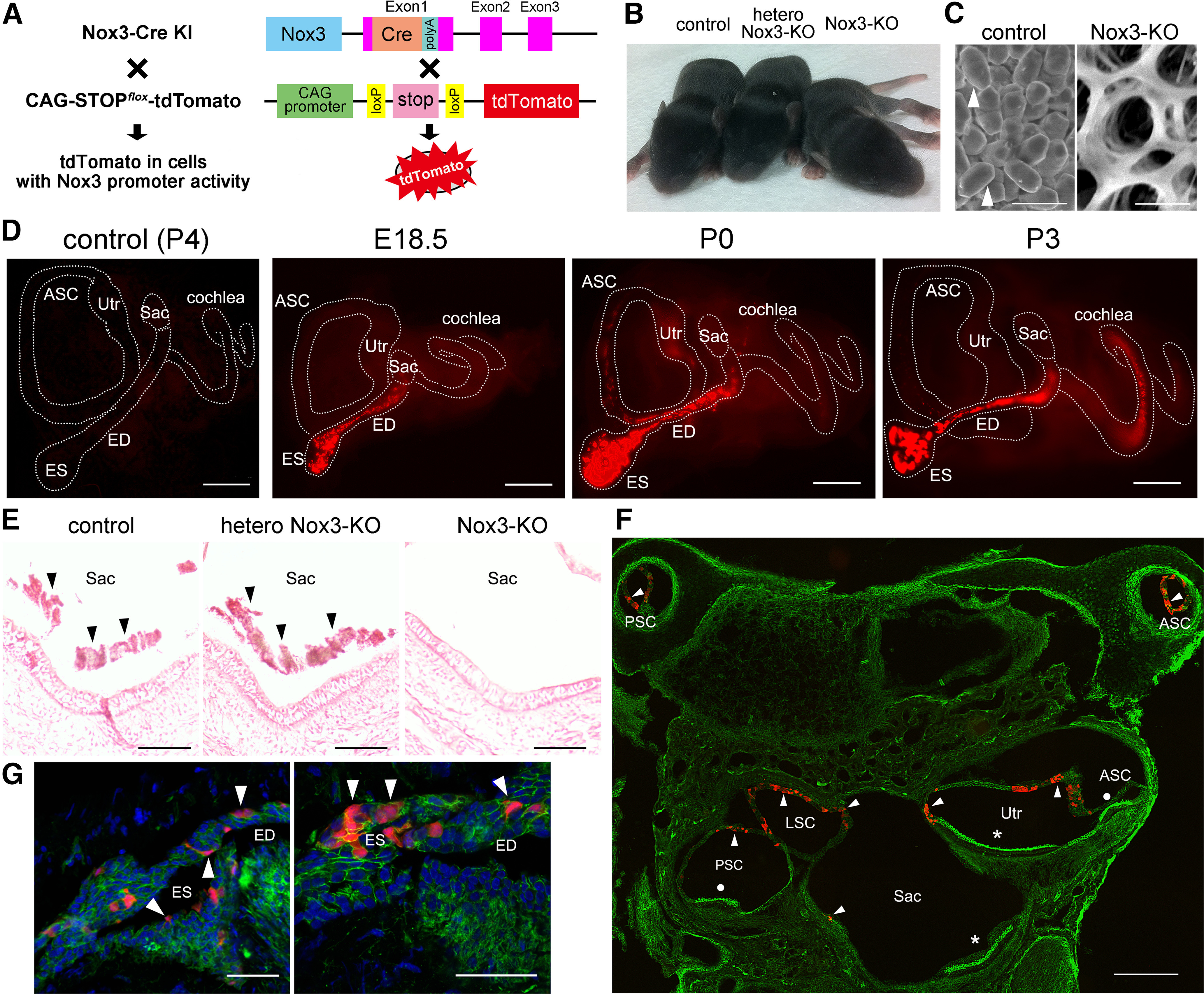
Generation of *Nox3-Cre* KI mice and Nox3 expression for otoconia formation. ***A***, Illustration showing the genetic construction of *Nox3* mutant mice (*Nox3-Cre* KI), in which *Cre* recombinase with a *poly A* sequence (*poly A*) is inserted in exon 1 of *Nox3*. *Nox3-Cre* KI mice were bred with *CAG-STOP^flox^-tdTomato* mice to obtain *Nox3-Cre*^−/−^*;tdTomato*^+/+^ (control), *Nox3-Cre*^+/−^*;tdTomato*^+/+^ (heterozygous [hetero] *Nox3*-KO) and *Nox3-Cre*^+/+^*;tdTomato*^+/+^ (*Nox3*-KO) lines. ***B***, Only *Nox3*-KO mice at P8 that display the tilted-head phenotype. ***C***, Utricles were obtained from P8 control and *Nox3*-KO mice for scanning electron microscopy. Arrowheads indicate otoconia in control mice that are absent in *Nox3*-KO mice. Scale bars, 5 μm. ***D***, Whole-mount inner ears from control mice at P4 and *Nox3-Cre*^+/+^*;tdTomato*^+/−^ mice at E18.5, P0, and P3. Fluorescence images represent tdTomato expression (red) in inner ears. ASC, Anterior semicircular canal; Utr, utricle; Sac, saccule. Scale bars, 500 μm. ***E***, Cryostat sections of the saccule obtained from P0 control, hetero *Nox3*-KO, and *Nox3*-KO mice, stained with H&E solution. Arrowheads indicate otoconia. Scale bars, 100 μm. ***F***, ***G***, Cryostat sections from P7 *Nox3-Cre*^+/+^*;tdTomato*^+/+^ mice were stained with Alexa-488-labeled phalloidin (green) with or without DAPI (blue). ***F***, Arrowheads indicate nonsensory tdTomato-positive cells (red) facing the lumens of the saccule, utricle, and semicircular canals. Asterisks indicate the maculae. Circles represent ampullae. PSC, Posterior semicircular canal; LSC, lateral semicircular canal. ***G***, ES and ED. Scale bars: ***G***, 50 μm; ***F***, 200 μm. Representative images of at least three independent experiments (***B–G***).

All animal experiments were approved (26-03-05 and 2020-06-04) and conducted in accordance with the Kobe University Animal Experimentation Regulations. We generated a mouse line in which the nuclear localization signal-tagged Cre recombinase encoding gene (*Cre*) fused to the poly-adenylation (poly A) sequence of SV40 (*SV40 poly A*) was inserted at the ATG site of exon 1 of *Nox3* on chromosome 17 (*Nox3-Cre* knock-in [KI]; see Fig. 1*A*). The targeting vector was constructed using PCR to contain *Cre*, *SV40 poly A*, and the *PGK* promoter (PGK) followed by a neomycin-resistant gene (*Neo*) flanked by flippase (FLP) recognition target sites (FRT). After confirming the plasmid by sequencing, the plasmid was linearized by SalI and the *short arm-Cre-SV40 poly A-FRT-PGK-Neo-FRT-long arm* was introduced into ES cells with a C57BL/6J background by electroporation. Homologous recombinant ES cell clones were identified by PCR and Southern blotting and were transferred to recipient C57BL/6J mice. Founder (F1) mice were screened by PCR using the following primer pairs: 5′-CTCTAGCTGTTGTCATCACTGAATC-3′ (outside of the short arm) and 5′-CTTCCTCGTGCTTTACGGTATC-3′ (in the Neo), resulting in a 4693 bp product (Unitech). The F1 mice were crossed with *CAG-FLP* mice (C57BL/6J) to remove the *Neo* cassette. F5 and later generations of *Nox3-Cre* KI mice were used for subsequent experiments. *CAG-STOP^flox^-tdTomato* (Ai9) reporter mice (C57BL/6J), in which the *ROSA26* region was used for transgene insertion, were purchased from Jackson Laboratory and backcrossed with *Nox3-Cre* KI mice to generate *Nox3-Cre*^−/−^*;tdTomato*^+/+^, *Nox3-Cre*^+/−^*;tdTomato*^+/−^, *Nox3-Cre*^+/−^*;tdTomato*^+/+^, *Nox3-Cre*^+/+^*;tdTomato*^+/−^, and *Nox3-Cre*^+/+^*;tdTomato*^+/+^ mice. In the latter four lines, the *Nox3*-promoter driven expression of Cre recombinase induces deletion of a stop codon sequence, leading to the expression of tdTomato (see Fig. 1*A*). The first (*Nox3-Cre*^−/−^*;tdTomato*^+/+^) and last (*Nox3-Cre*^+/+^*;tdTomato*^+/+^) lines were used as control and *Nox3*-KO mice, respectively. The offspring of these mice were genotyped by PCR using the following primer pairs: 5′-CTTGGCACTAAGTCCTTGATTAG-3′ and 5′-CAGTGAAACAGCATTGCTGTC-3′ for *Nox3-Cre*; 5′-CTGTTCCTGTACGGCATGG-3′ and 5′-GGCATTAAAGCAGCGTATCC-3′ for positive integration of *tdTomato* (196 bp); 5′-AAGGGAGCTGCAGTGGAGTA-3′ and 5′-CCGAAAATCTGTGGGAAGTC-3′ for negative integration of *tdTomato* (297 bp). Mice were housed in specific pathogen-free animal care facilities using an individually ventilated cage system (Techniplast) and provided food and water *ad libitum*. The animal care facility was maintained on a 14 h light/10 h dark cycle at 23 ± 2°C and 50 ± 10% humidity. Both male and female mice were used in the analyses unless otherwise indicated. Age- and sex-matched WT mice were also used as controls. Mice <1 week of age were not differentiated based on sex.

##### Antibodies and chemicals

A Myosin-VIIa polyclonal antibody (Ab) (25-6790, Proteus Biosciences, RRID: AB_10015251, 1/400), βIII-tubulin monoclonal Ab (Cell Signaling Technology, RRID: AB_10694505, 1/200), CtBP2 monoclonal Ab (BD Biosciences, catalog #612044, RRID:AB_399431; 1:200), AlexaFluor-488-labeled phalloidin (Thermo Fisher Scientific, RRID:AB_2315147, 1/500), and AlexaFluor-488-labeled secondary Ab (Thermo Fisher Scientific, RRID:AB_143165, 1/2000) were used for imaging analyses. H&E solutions were purchased from Muto Pure Chemicals. CDDP was purchased from Fujifilm Wako Pure Chemical.

##### Auditory brainstem response (ABR) measurement

For ABR measurements (Ueyama et al., 2016), mice were anesthetized with a mixture of 0.3 mg/kg medetomidine, 4.0 mg/kg midazolam, and 5.0 mg/kg butorphanol administered by intraperitoneal injection, and stainless-steel needle electrodes were placed at the vertex and ventrolateral to the left and right ears. Electroencephalographic recordings were performed with BioSigRP Software and the TDT System 3 (Tucker-Davis Technologies) to generate tone-burst stimulation at 8, 16, 24, or 32 kHz. ABR waveforms were recorded for 12.8 ms at a sampling rate of 40,000 Hz by using 50-5000 Hz bandpass filter settings. Waveforms from 500 stimuli were averaged. ABR thresholds were defined by decreasing the sound intensity in 5 dB steps from 90 dB SPL until the lowest sound intensity level resulting in a recognizable ABR wave pattern, which was mainly judged by the recognition of wave III, was reached. When there was no response to the stimulation at 90 dB, the threshold was considered to be 100 dB.

##### Age-related, cisplatin-induced, and noise-induced hearing loss

For the evaluation of ARHL, 1-, 2-, and 6-month-old control (*Nox3-Cre*^−/−^*;tdTomato*^+/+^), *Nox3-Cre*^+/−^*;tdTomato*^+/+^, and *Nox3-Cre*^+/+^*;tdTomato*^+/+^ (*Nox3*-KO) mice were used.

The protocols for experiments using CDDP treatments and ABR measurement are shown in Figure 5*A*. CDDP was dissolved in saline and intraperitoneally administered at 5 mg/kg for 6 consecutive days to 1-, 2-, and 6-month-old control, *Nox3-Cre*^+/−^*;tdTomato*^+/+^ (hetero *Nox3*-KO), and *Nox3-Cre*^+/+^*;tdTomato*^+/+^ (*Nox3*-KO) mice. ABR was measured twice: before CDDP treatment at day 0 and before fixation at day 7. We set the endpoint of the experiments, which was defined as a decrease of >1/4 the body weight at day 4 with daily body weight measurements.

The protocol for experiments with NE and ABR measurements is shown in Figure 9*A*. Two-month-old control, *Nox3-Cre*^+/−^*;tdTomato*^+/+^, and *Nox3-Cre*^+/+^*;tdTomato*^+/+^ (*Nox3*-KO) mice were anesthetized as described and exposed to octave-band noise at 120 dB SPL centered at 8 kHz for 3 h inside a sound chamber. The condition of NE to induce a permanent threshold shift was determined based on our previous report (Morioka et al., 2018). Each animal was placed in a cage in the sound chamber, which was fitted with a speaker (300 HT; Fostex) driven by a noise generator (SF-06; Rion) and power amplifier (DAD-M100proHT; Flying Mole). To ensure the uniformity of the stimulus, we calibrated and measured the sound levels with a sound level meter (NL-26: Rion) positioned at the level of the animal's head. The ABRs were measured immediately before NE, and then ABR was sequentially measured immediately after NE on day 0 (all these procedures were performed during one anesthesia session with the aforementioned dose) and at days 2 and 7 after NE. Hearing deterioration because of NE was evaluated by measuring the ABR threshold shift, calculated by the differences in ABR threshold before and after NE.

##### Histochemistry

Whole-mount inner ears or cochleae (with bone), surface preparations of cochleae (without bony structures), and 12 μm cryostat sections of dissected cochleae, all of which were first fixed with 4% PFA in 0.1 m PB, pH 7.4, were examined as previously described (Ueyama et al., 2014; Morioka et al., 2020). Samples for surface preparations and cryostat sections were decalcified in 0.12 m EDTA for 1 week at 4°C or for 2 d at 23°C. After permeabilization with PBS containing 0.3% Triton X-100 (PBS-0.3T) and blocking with 5% fat-free BSA, fixed tissues were incubated with primary antibodies for 2 h at 23°C in PBS-0.03T containing 3% fat-free BSA, followed by AlexaFluor-488-labeled secondary antibodies for 1 h at 23°C. After permeabilization with PBS-0.3T, fixed tissues were also incubated with AlexaFluor-488-labeled phalloidin with DAPI as a nuclear counterstain for 1 h at 23°C. Stained tissues were mounted in Prolong anti-fade (Thermo Fisher Scientific) with a coverslip and observed under an LSM700 confocal microscope (Carl Zeiss). To more clearly distinguish HCs and SCs in the surface preparation (not like in cryostat sections, which allow for spatial determination), immunostaining for myosin-VIIa, a marker for HCs, was performed (except in Figs. 4*C*, 5*C*). In cases of otoconia observation, inner ears were fixed with 4% PFA in HEPES buffer, pH 7.4, and stained with H&E solutions.

##### Hair cell and presynaptic ribbon counts

Myosin-VIIa immunostaining (or phalloidin staining) was used for estimating OHC and IHC counts. We acquired images at five different regions in the apical, middle, and basal turn (390°, 450°, and 510°) from control (*Nox3-Cre*^−/−^*;tdTomato*^+/+^) and *Nox3-Cre*^+/+^*;tdTomato*^+/+^ (*Nox3*-KO) mice.

Cochleae of 6-month-old control (*Nox3-Cre*^−/−^*;tdTomato*^+/+^) and *Nox3-Cre*^+/+^*;tdTomato*^+/+^ (*Nox3*-KO) mice were fixed with 4% PFA in 0.1 m PB for 2 h at 23°C, followed by decalcification in 0.12 m EDTA for 2 d at 23°C. Tissues were dissected into four half-turns and hook portions and immunostained with CtBP2 to detect presynaptic ribbons using phalloidin counterstaining. Samples were imaged and stacked in 0.40 µm intervals at 300 µm-spaced regions between 360° to 450° from the apex on the cochlear spiral, which corresponds to nearly 24-32 kHz, using a confocal microscope (LSM900) with an oil-immersion 63× objective (1.4 numerical aperture), as described previously (Ninoyu et al., 2020). IHCs with stereocilia and ribbon synapses were manually counted, and the average number of ribbon synapses per IHC was then calculated.

##### TUNEL assay

Apoptosis detection in cochleae was performed using an ApopTag Fluorescein In Situ Apoptosis Detection Kit S7110 (Merck Millipore) according to the manufacturer's protocol. Samples were fixed with 4% PFA in 0.1 m PB, pH 7.4, and postfixed with methanol at −20°C for 10 min. After permeabilization with 20 μg/ml proteinase K for 15 min, surface preparations or cryostat sections of samples were incubated in equilibration buffer for 10 min and then terminal deoxynucleotidyl transferase and dNTP-digoxigenin were added to the samples and incubated in a humidified chamber at 37°C for 1 h. The reaction was stopped, and samples were incubated with anti-digoxigenin fluorescein solution for 30 min at 23°C. Finally, samples were incubated with DAPI as a nuclear counterstain for 30 min before imaging.

##### DNA microarray

DNA microarray was performed as previously described (Ueyama et al., 2020). Briefly, total RNA of the inner ear was extracted from three littermates of each group (P4 *Nox3-Cre*^−/−^*;tdTomato*^+/+^ = *WT* versus (vs) *Nox3-Cre*^+/−^*;tdTomato*^+/+^ = hetero *Nox3*-KO) using NucleoSpin RNA (Macherey-Nagel). Gene expression profiles were examined using a mouse oligo chip (24k), 3D-Gene (Toray Industries), and the signal strength of each gene was presented after global normalization.

##### Experimental design and statistical analysis

To achieve a statistical power of 0.8 with a 50% effect size with means and SDs, sample sizes were calculated using a power analysis based on variances of the ABR threshold and HC and presynaptic ribbon counts reported previously (Ueyama et al., 2016; Ninoyu et al., 2020). As several experiments had considerably large effect sizes, sample sizes were adjusted accordingly. All data are presented as the mean ± SEM. For the comparisons of two groups, an unpaired two-tailed Student's *t* test was used. The *t* values and the degrees of freedom (df) are shown as *t*_(df)_ in Results. For comparisons of more than two groups, one-way or two-way ANOVA was performed, followed by *post hoc* Tukey's test for pairwise group differences. The *F* ratio, DFn, and DFd were presented as *F*_(DFn, DFd)_ in Results, where DFn stands for degrees of freedom of the numerator and DFd for degrees of freedom of the denominator. Statistical analyses were performed using Prism 7.0 software (GraphPad); *p* < 0.05 was considered significant. For transparency, all individual data points are included in the figures. All statistical details, including the exact *n* and *p* values, and the statistical tests performed can be found in the figure legends. CIHL and ARHL studies were performed as cross-sectional studies. For comparisons of Nox3 involvement in CIHL, ARHL, and NIHL, the ratios of the mean ABR thresholds were used in accordance with previous reports (Friedrich et al., 2011; Park et al., 2013).

## Results

### Generation of *Nox3-Cre* KI and *Nox3-Cre*^+/+^*;tdTomato*^+/+^ (*Nox3-KO*) mice

To identify where Nox3 is expressed in the inner ear, we generated *Nox3-Cre* KI mice using a homologous recombination method (Fig. 1*A*). *Nox3-Cre* KI mice were intercrossed with *CAG-STOP^flox^-tdTomato* reporter mice (hereafter, the offspring are referred to as *Nox3-Cre*^+/−^*;tdTomato*^+/^^−^), in which tdTomato was expressed in cells under a functionally active *Nox3* promoter. *Nox3-Cre*^+/−^*;tdTomato*^+/^^−^ mice were further crossed with *Nox3-Cre*^+/−^*;tdTomato*^+/^^−^ to obtain *Nox3-Cre*^−/−^*;tdTomato*^+/+^, *Nox3-Cre*^+/−^*;tdTomato*^+/+^, *Nox3-Cre*^+/+^*;tdTomato*^+/−^, and *Nox3-Cre*^+/+^*;tdTomato*^+/+^ populations (Fig. 1*A*). In the latter three genetic backgrounds, exon 1 of *Nox3* was replaced with *Cre* at one and both alleles, respectively, resulting in heterozygous *Nox3*-KO (hetero *Nox3*-KO) and homozygous *Nox3*-KO (*Nox3*-KO) mice. Consistent with a previous report (Paffenholz et al., 2004), *Nox3*-KO mice, but not the control (*Nox3-Cre*^−/−^*;tdTomato*^+/+^) or hetero *Nox3*-KO mice, displayed a “tilted head” phenotype. An otoconia defect was observed in *Nox3*-KO mice that was identified by scanning electron microscopy (Fig. 1*B*,*C*).

### Nox3 expression in the ES, ED, vestibule, and semicircular canals

To detect cells producing Nox3-derived ROS required for otoconia biosynthesis, we examined tdTomato-positive cells (as an indicator of Nox3-expressing cells) in *Nox3-Cre*^+/+^*;tdTomato*^+/−^ mice at embryonic day 18.5 (E18.5), postnatal day 0 (P0), and P3, under a fluorescence microscope. In whole-mount inner ears, strong tdTomato fluorescence was observed in the ES and ED from E18.5, and weak tdTomato fluorescence was observed in semicircular canals and the vestibule (Fig. 1*D*) at P0, as well as in the cochlea, where expression was predominantly in the basal turn at P3 (Fig. 1*D*). This specific tdTomato expression was not observed in control mice (*Nox3-Cre*^−/−^*;tdTomato*^+/+^); moreover, *Nox3*-KO mice showed normal morphologic development of the inner ear (Fig. 1*D*). Control (*Nox3-Cre*^−/−^*;tdTomato*^+/+^) and hetero *Nox3*-KO mice had otoconia at the saccule at P0, whereas *Nox3*-KO (*Nox3-Cre*^+/+^*;tdTomato*^+/+^) mice did not (Fig. 1*E*). These results suggest that Nox3-derived ROS in the ES and ED are required for otoconia formation.

Next, we examined cell types expressing Nox3 in the ES, ED, vestibule, and semicircular canals using P7 *Nox3-Cre*^+/+^*;tdTomato*^+/+^ mice. Cryostat sections revealed that nonsensory epithelial cells, facing the lumens of the ES, ED, vestibule, and semicircular canals, but not HCs (sensory epithelia) of the maculae or ampulla, exhibited tdTomato fluorescence (Fig. 1*F*,*G*).

### Time-dependent increase in Nox3 expression in the cochlea

**Figure 2.**
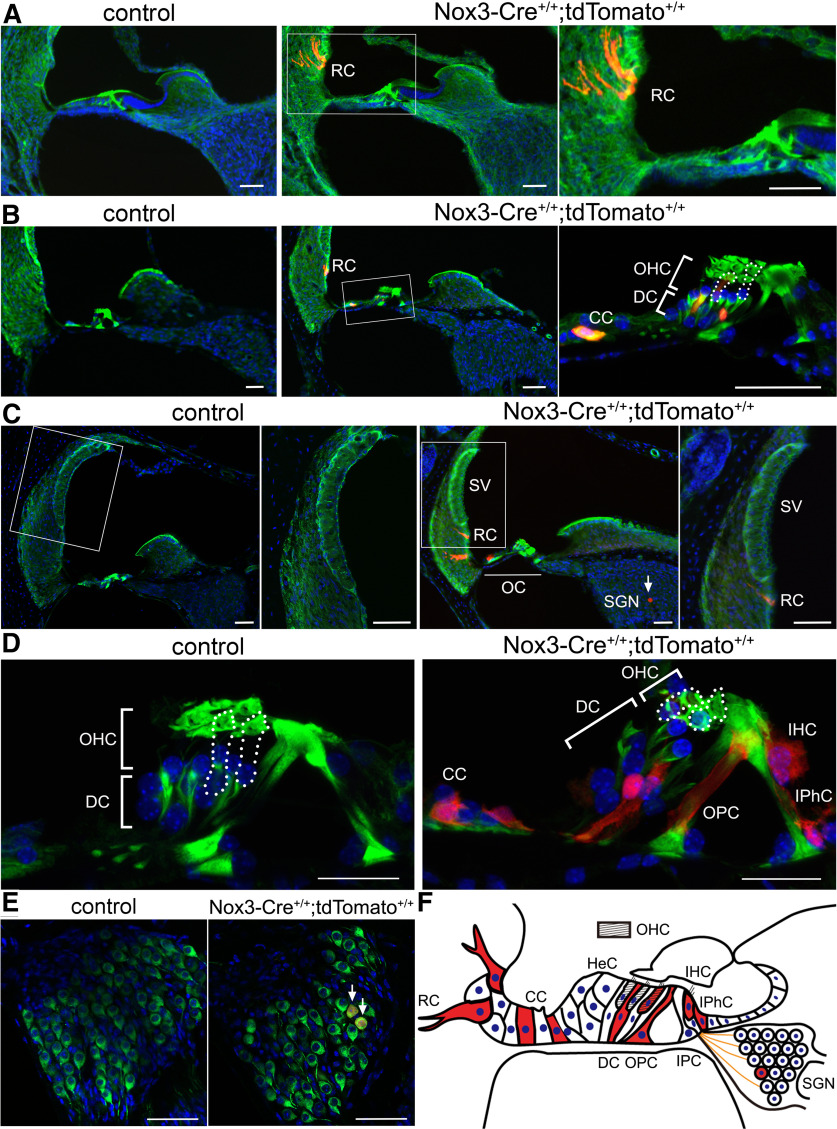
Time-dependent Nox3 expression in cochleae. Cochlear cryostat sections from (***A***) P7, (***B***) 2-month-old, and (***C–E***) 12-month-old control (*Nox3-Cre*^−/−^*;tdTomato*^+/+^) and *Nox3-Cre*^+/+^*;tdTomato*^+/+^ mice stained with Alexa-488-labeled phalloidin (green, ***A-D***) or βIII-tubulin (green, ***E***) with DAPI (blue). Fluorescence images of tdTomato-positive cells (red). Outlined cells by dots indicate OHCs. ***A***, Right, Magnified image of the area indicated in a rectangle in the middle panel. tdTomato-positive RCs are present in the lateral wall of the cochlea. ***B***, tdTomato-positive cells in the OC. Right, Magnified image of the area in a rectangle in the middle panel. OHCs, CCs, and DCs, which are SCs, are positive for tdTomato. ***C***, Rectangles represent magnified images of the lateral wall. Cells in the SV are negative for tdTomato. Arrow indicates tdTomato-positive SGN. ***D***, Increased number of tdTomato-positive cells in the OC. In addition to CCs and DCs, SCs, such as the OPCs and IPhCs, are positive for tdTomato. tdTomato-positive IHCs are also present. ***E***, Magnified images of the SG show SGNs double-positive for tdTomato and βIII-tubulin (arrows). Representative images were taken from at least three independent experiments. Scale bars: ***A-C***, ***E***, 50 μm; ***D***, 25 μm. ***F***, Illustration showing tdTomato-positive cells (red) in the cochlea. OC has two types of HCs: IHCs and OHCs (oblique lines). HCs are surrounded by SCs, such as CCs, DCs, OPCs, IPCs, IPhCs, and HeCs. RCs and SGNs reside in the lateral wall and the SG, respectively.

To spatially and morphologically identify cell types expressing Nox3 in the cochlea, we examined cryostat sections from *Nox3-Cre*^+/+^*;tdTomato*^+/+^ mice at P7, 2 months, and 12 months of age. We observed tdTomato fluorescence in root cells (RCs) in the lateral wall of the cochlea at P7 (Fig. 2*A*). At 2 months, tdTomato-positive cells included SCs, such as DCs and CCs, and OHCs in addition to RCs (Fig. 2*B*). At 12 months, tdTomato expression further increased in SCs, such as DCs, CCs, OPCs, and IPhCs, and was observed in SGNs in *Nox3-Cre*^+/+^*;tdTomato*^+/+^ but not in control mice (Fig. 2*C*,*D*). Additionally, tdTomato was observed in IHCs at 12 months, but not P7 or 2 months (Fig. 2*D*), and tdTomato-positive cells in the spiral ganglion (SG) were positive for βIII-tubulin (Fig. 2*E*). These results suggest that Nox3 expression in these cell types in the cochlea (Fig. 2*F*) increased in a time-dependent manner.

### Age-related increase in Nox3 expression in the OC is accompanied by hearing loss

**Figure 3.**
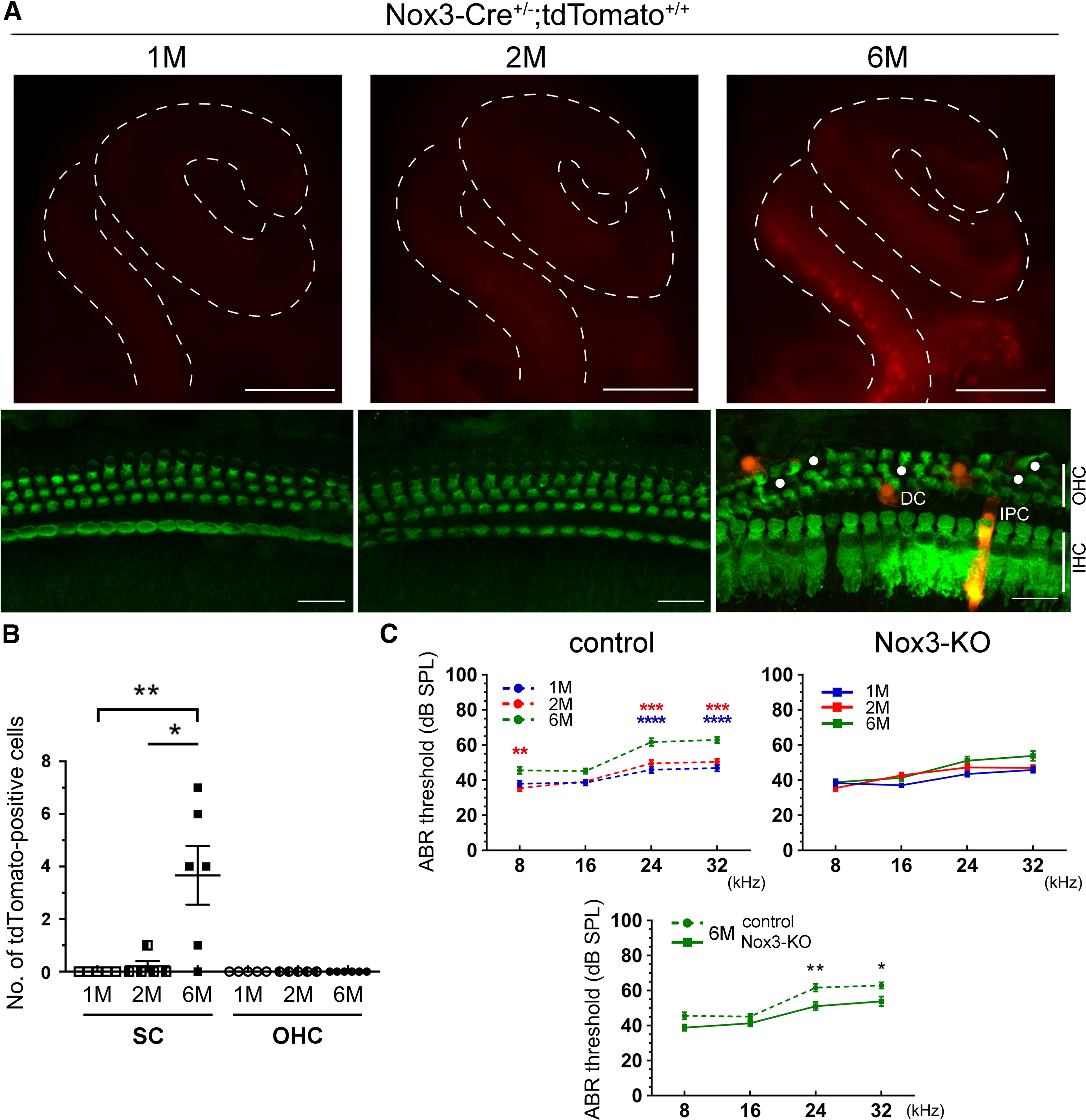
Increased cochlear Nox3 expression and hearing loss in aged mice. ***A***, Whole-mount samples (top panels) showing tdTomato expression (red) in cochleae at 1-, 2-, and 6-month-old (M) *Nox3-Cre*^+/−^*;tdTomato*^+/+^ mice. Surface preparations at the basal turn (510°; bottom panels) stained against myosin-VIIa (green). Circles represent OHC loss. Representative images of three experiments. Scale bars: top, 500 μm; bottom, 20 μm. ***B***, Graph of the number of tdTomato-positive SCs and OHCs in surface preparations at the basal turn (510°, 1 month, *n* = 5; 2 months, *n* = 5; 6 months, *n* = 6, **p = 0.0095 for 1 month vs 6 months and *p = 0.0137 for 2 months vs 6 months by one-way ANOVA with Turkey's *post hoc* test). ***C***, Age-related pure tone-burst (8, 16, 24, and 32 kHz) ABR thresholds (dB SPL, mean ± SEM) in control (1 month, *n* = 13; 2 months, *n* = 12; 6 months, *n* = 12) and *Nox3*-KO mice (1 month, *n* = 12; 2 months, *n* = 10; 6 months, *n* = 11). Age-related hearing loss is reduced in *Nox3*-KO mice. ****p < 0.0001, ***p = 0.0002 (1 month vs 6 months and 2 months vs 6 months at 24 kHz). ****p < 0.0001, ***p = 0.0001 (1 month vs 6 months and 2 months vs 6 months at 32 kHz). Top left, **p = 0.0050 (2 months vs 6 months at 8 kHz). Bottom, **p = 0.0080 (24 kHz), *p = 0.0404 (32 kHz) at 6 months (control vs *Nox3*-KO). Two-way ANOVA with Turkey's *post hoc* test.

To further examine how Nox3 expression in the cochlea changes over time, we used whole-mount cochleae and surface preparations of the OC from *Nox3-Cre*^+/−^*;tdTomato*^+/+^ mice at 1, 2, and 6 months of age. At 1 and 2 months, almost no tdTomato expression was observed in whole-mount cochleae; however, tdTomato expression with OHC loss was observed predominantly at the basal turn (510°) of the cochlea at 6 months (Fig. 3*A*). In OC surface preparations at the basal turn (510°), the number of tdTomato-positive SCs, but not OHCs, significantly increased between 1 month and 6 months (0.00 ± 0.00 cells vs 3.67 ± 1.12 cells, *p* = 0.0095, *F*_(2,13)_ = 5.000) and between 2 months and 6 months (0.20 ± 0.20 cells vs 3.67 ± 1.12 cells, *p* = 0.0137, *F*_(2,13)_ = 4.727; Fig. 3*B*).

To elucidate the association between Nox3 expression and ARHL, we evaluated ABR in 1-, 2-, and 6-month-old control and *Nox3*-KO mice. Six-month-old control mice showed a significant increase in the ABR threshold at 8, 24, and 32 kHz (2 months vs 6 months at 8 kHz, *p* = 0.0050, *F*_(1,192)_ = 5.624, 1 month vs 6 months and 2 months vs 6 months at 24 kHz, *p* < 0.0001 and *p* = 0.0002, *F*_(1,192)_ = 8.823 and *F*_(1,192)_ = 6.657, and 1 month vs 6 months and 2 months vs 6 months at 32 kHz, *p* < 0.0001 and *p* = 0.0001, *F*_(1,192)_ = 8.985 and *F*_(1,192)_ = 6.886; Fig. 3*C*, top left), whereas *Nox3*-KO mice demonstrated no such increase (Fig. 3*C*, top right). The ABR thresholds at high-frequency sounds (24 and 32 kHz) were significantly higher in control mice than in *Nox3*-KO mice at 6 months (24 kHz, *p* = 0.0080, *F*_(1,176)_ = 5.169 and 32 kHz, *p* = 0.0404, *F*_(1,176)_ = 4.444; Fig. 3*C*, bottom). These data suggest that increased Nox3 expression in the OC, such as SCs, is one of the factors that cause ARHL at 6 months.

**Figure 4.**
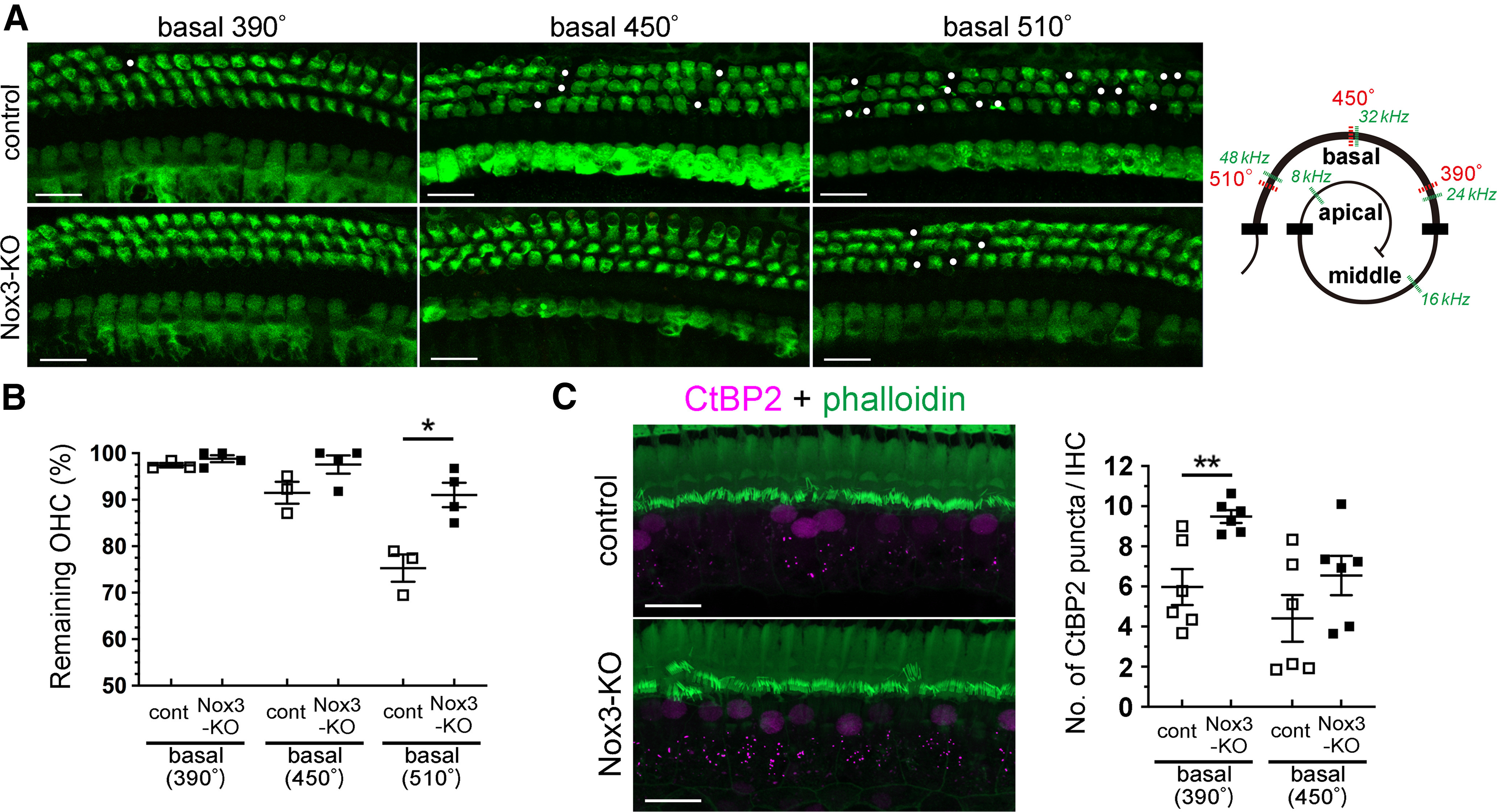
Ameliorated OHC and ribbon synapse loss in aged *Nox3*-KO mice. ***A***, ***B***, Surface preparations of the OC at the basal turn (390°, 450°, and 510°) from 6-month-old control and *Nox3*-KO mice were stained using a myosin-VIIa antibody (green). OHC loss was detected using confocal microscopy and the percentages of the remaining OHCs are graphically represented. Circles represent OHC loss. *p = 0.0106 (510°, control vs *Nox3*-KO; *n* = 3 and *n* = 4, respectively) by Student's *t* test. Scale bars, 20 μm. The illustration shows the regions, degrees (°), and frequencies (kHz) of the cochlea. ***C***, Surface preparations of the OC at the basal turn from 6-month-old control and *Nox3*-KO mice were stained with CtBP2 and Alexa-488-labeled phalloidin, and confocal microscopic images were obtained. Graph represents the number of CtBP2-positive puncta per IHC at the basal turn (390° and 450°). *n* = 6. **p = 0.0042 (390°, control vs *Nox3*-KO) by Student's *t* test. Scale bars, 20 μm.

In accordance with histologic analysis, control mice at 6 months exhibited OHC loss at the basal turn with apical-to-basal progression, consistent with the high-frequency hearing loss (Fig. 4*A*). In contrast, OHC loss in *Nox3*-KO mice was significantly lower at the basal turn (510°) compared with that in control mice (*p* = 0.0106, *t*_(5)_ = 3.973; Fig. 4*A*,*B*). The percentages of remaining OHCs at 510° in control and *Nox3*-KO mice were 75.3 ± 2.9% vs 91.0 ± 2.6% (Fig. 4*B*). To further examine the mechanism of OHC loss induced by Nox3-derived ROS, presynaptic ribbons were evaluated at the basal turn (390° and 450°), wherein no significant differences were observed in remaining OHCs between 6-month-old control and *Nox3*-KO mice. The number of ribbon synapses per IHC labeled by CtBP2 at the basal turn (390°) was significantly lower in control mice than in *Nox3*-KO mice (*p* = 0.0042, *t*_(10)_ = 3.690; Fig. 4*C*), suggesting that Nox3-derived ROS affect ribbon synapses before OHC loss.

### Increased Nox3 expression at the OC basal turn: the main target of CDDP ototoxicity

**Figure 5.**
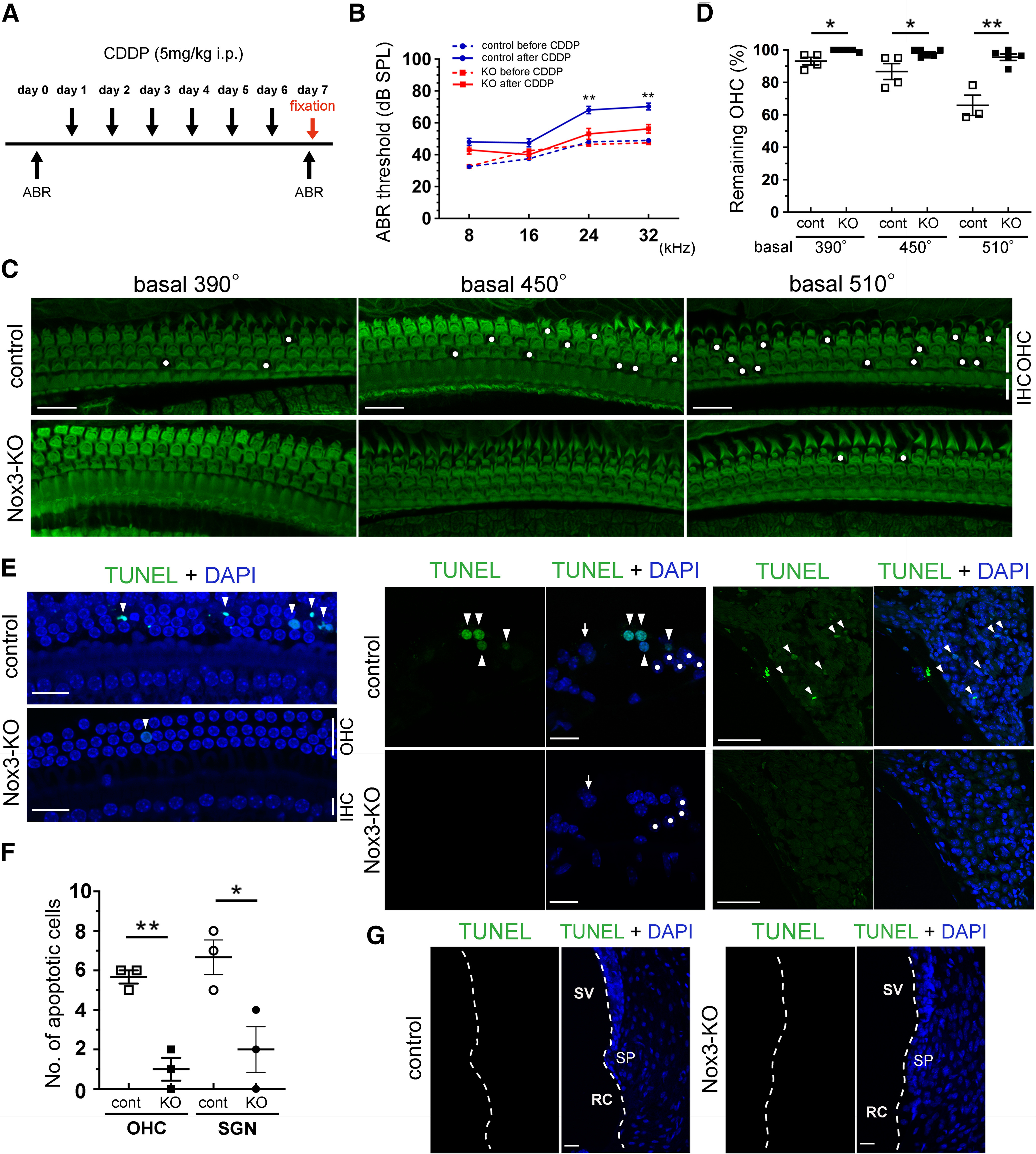
Ameliorated cisplatin (CDDP)-induced hearing loss and apoptosis in *Nox3*-KO mice. ***A***, ABR measurements and CDDP treatment administered to 2-month-old control and *Nox3*-KO mice intraperitoneally at 5 mg/kg for 6 consecutive days. ***B***, Pure tone-burst (8, 16, 24, and 32 kHz) ABR thresholds (dB SPL, mean ± SEM) in control and *Nox3*-KO mice before and after CDDP treatment (*n* = 8). **p = 0.0023 (24 kHz after CDDP, control vs KO); **p = 0.0055 (32 kHz after CDDP, control vs KO); two-way ANOVA with Turkey's *post hoc* test. ***C***, ***D***, Surface preparations of the OC at the basal turn (390°, 450°, and 510°) from control and *Nox3*-KO mice at day 7, stained with Alexa-488-labeled phalloidin (green). OHC loss was detected using confocal microscopy, and the percentages of the remaining OHCs were graphed. Circles represent OHC loss. *p = 0.0156 (390°, control vs KO; *n* = 4 and *n* = 5, respectively); *p = 0.0217 (450°, control vs KO; *n* = 4 and *n* = 6, respectively); **p = 0.0014 (510°, control vs KO; *n* = 3 and *n* = 5, respectively); Student's *t* test. Scale bars, 20 μm. ***E–G***, Surface preparations of the basal turn (450°) (***E***, left), and cryostat sections of the OC (***E***, middle), SG (***E***, right), and lateral wall (***G***) from control and *Nox3*-KO mice at day 7 for TUNEL (green) and DAPI (blue) staining. Arrowheads indicate TUNEL-positive OHCs and SGNs. Circles represent DCs. Scale bars, 20 μm. ***F***, Graphs represent quantification of the number of TUNEL-positive OHCs in surface preparations (left, *n* = 3, **p = 0.0022) and SGNs in cryostat sections (right, *n* = 3, *p = 0.0325) by Student's *t* test. ***G***, No TUNEL-positive IHCs (arrows), RCs, or cells in the SV were observed. SP, Spiral prominence.

Previous studies have reported that Nox3 is the main source of CDDP-induced ROS production (Mukherjea et al., 2006, 2010). Here, we assessed CIHL in 2-month-old control and *Nox3*-KO mice by measuring the ABR to pure tone-burst stimuli (8, 16, 24, and 32 kHz) using the protocol shown in Figure 5*A*. After CDDP treatment, control mice exhibited significantly deteriorated ABR thresholds at 24 and 32 kHz compared with those for *Nox3*-KO mice (24 kHz, *p* = 0.0023, *F*_(1,120)_ = 5.727 and 32 kHz, *p* = 0.0055, *F*_(1,120)_ = 5.369; Fig. 5*B*). Consistent with the ABR data, control mice showed OHC loss at the basal turn with a basal-to-apical gradient, whereas OHC loss in *Nox3*-KO mice was significantly lower in all three segments of the basal turn (390°, 450°, and 510°) than that in control mice (Fig. 5*C*,*D*). The percentages of remaining OHCs in control and *Nox3*-KO mice were 93.1 ± 2.3% vs 99.7 ± 0.3% (390°, *p* = 0.0156, *t*_(7)_ = 3.174), 86.7 ± 4.9% vs 98.1 ± 0.9% (450°, *p* = 0.0217, *t*_(8)_ = 2.844), and 65.9 ± 6.3% vs 95.6 ± 2.0% (510°, *p* = 0.0014, *t*_(6)_ = 5.587), respectively (Fig. 5*D*). Previous studies have reported that CDDP induces apoptosis in OHCs and SGNs (Mukherjea et al., 2010) (predominantly in OHCs) (Benkafadar et al., 2017); therefore, we examined apoptosis using the TUNEL assay in our mouse model. A significantly larger number of TUNEL-positive OHCs was observed at the basal turn (450°) in control mice than that in *Nox3*-KO mice (*p* = 0.0022, *t*_(4)_ = 7.000; Fig. 5*E*,*F*). Similarly, TUNEL-positive SGNs at the basal turn were significantly fewer in *Nox3*-KO mice than those in control mice (*p* = 0.0325, *t*_(4)_ = 3.212; Fig. 5*E*,*F*). No TUNEL-positive cells were observed in the lateral wall of the cochlea, including RCs and the stria vascularis (SV), in both control and *Nox3*-KO mice (Fig. 5*G*). Together, these data indicate that apoptosis of OHCs and SGNs, which causes CIHL, is attenuated in *Nox3*-KO mice.

**Figure 6.**
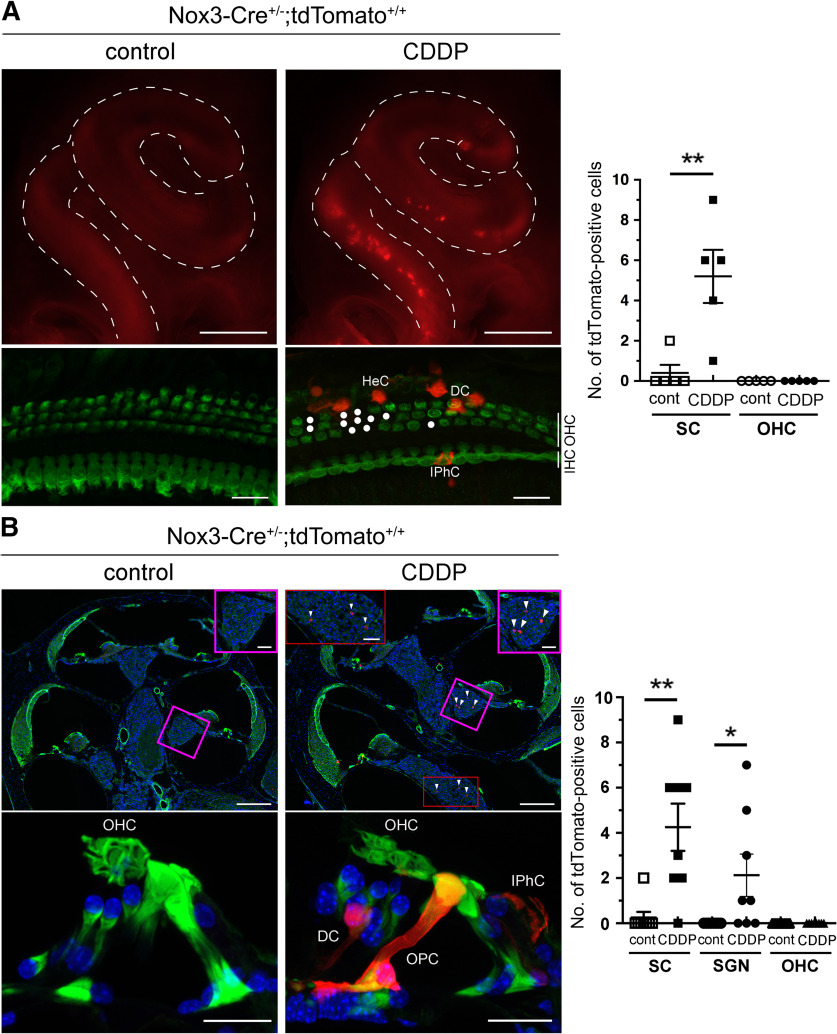
Increase in Nox3 expression at the basal turn of cochleae after cisplatin (CDDP). Inner ears of 2-month-old NaCl (control) or CDDP-treated *Nox3-Cre*^+/−^*;tdTomato*^+/+^ mice were fixed and used for (***A***) whole-mount fluorescence imaging (top panels), which showed enhanced tdTomato expression (red) in the cochlea after CDDP treatment. Fluorescence images of the surface preparations at the basal turn (510°, bottom panels) were stained using a myosin-VIIa antibody (green). Circles represent OHC loss. Images are representative of three experiments. Scale bars: top panels, 500 μm; bottom panels, 20 μm. Graphs represent the numbers of tdTomato-positive SCs and OHCs in surface preparations at the basal turn (510°). *n* = 5; **p = 0.0083 (Student's *t* test). ***B***, Cochlear cryostat sections were stained with Alexa-488-labeled phalloidin (green) and DAPI (blue). Arrowheads indicate tdTomato-positive SGNs. Insets, Magnified images of the areas shown in squares and rectangle on the main images. Scale bars, 50 μm. Graphs of the number of tdTomato-positive SCs, SGNs, and OHCs per three cochlear cryostat sections, respectively (*n* = 8; **p = 0.0023, *p = 0.0392 by Student's *t* test). Scale bars: top panels, 200 μm; bottom panels, 20 μm.

Next, we examined how tdTomato expression in the OC is affected by CDDP treatment in 2-month-old *Nox3-Cre*^+/−^*;tdTomato*^+/+^ mice. In the CDDP-treated group, tdTomato fluorescence increased predominantly at the basal turn of the cochlea in whole-mount cochlea samples (Fig. 6*A*, top panels), as well as in the SCs, which was accompanied by OHC loss in surface preparations at the basal turn (510°, *p* = 0.0083, *t*_(8)_ = 3.482; Fig. 6*A*, bottom panels). However, tdTomato-positive OHCs were not observed in these samples. In cryostat sections, the number of tdTomato-positive SCs and SGNs was significantly increased in the CDDP-treated group compared with that in the untreated control (SCs: *p* = 0.0023, *t*_(14)_ = 3.713; SGNs: *p* = 0.0392, *t*_(14)_ = 2.275; Fig. 6*B*). We counted the number of tdTomato-positive cells on one surface preparation (Fig. 6*A*) and in three cryostat sections (Fig. 6*B*; Table 1). Intriguingly, no tdTomato-positive OHCs were observed with or without CDDP treatment (Fig. 6*A*,*B*; Table 1), in contrast to the rare tdTomato-positive IHCs identified following CDDP treatment (Table 1). These data suggest that increased Nox3 expression at the basal turn of the OC is the main target of CDDP ototoxicity. However, why tdTomato expression was not present in OHCs from *Nox3-Cre*^+/−^*;tdTomato*^+/+^ mice was unclear from these experiments.

**Table 1.** The number of tdTomato-positive cells in *Nox3-Cre*^+/−^*;tdTomato*^+/+^ cochleae*^a^*

Cell type	1 month		2 months		6 months	
	Control	CDDP	Control	CDDP	Control	CDDP
SC	0.00 ± 0.00	3.75 ± 1.03	0.25 ± 0.25	4.25 ± 1.05	3.00 ± 1.23	5.33 ± 1.76
OHC	0.00 ± 0.00	0.00 ± 0.00	0.00 ± 0.00	0.00 ± 0.00	0.00 ± 0.00	0.00 ± 0.00
IHC	0.00 ± 0.00	0.00 ± 0.00	0.00 ± 0.00	0.37 ± 0.26	0.00 ± 0.00	0.67 ± 0.67
SGN	0.00 ± 0.00	1.00 ± 0.71	0.00 ± 0.00	2.13 ± 0.93	0.75 ± 0.75	2.00 ± 1.16
Total	0.00 ± 0.00	4.75 ± 1.65	0.25 ± 0.25	6.75 ± 1.78	3.75 ± 1.44	8.00 ± 2.00

*^a^*Data are mean ± SEM. *Nox3-Cre*^+/−^*;tdTomato*^+/+^ mice treated with either NaCl (control) or cisplatin (CDDP) were fixed at 1, 2, and 6 months of age. The numbers of tdTomato-positive cells per three cochlear cryostat sections were counted, and the means were obtained (*n* = 4 for 1 month, *n* = 8 for 2 months, and *n* = 4 and *n* = 3 in control and cisplatin [CDDP], respectively, for 6 months).

### CDDP-induced Nox3 expression in SCs and OHCs associated with OHC loss

**Figure 7.**
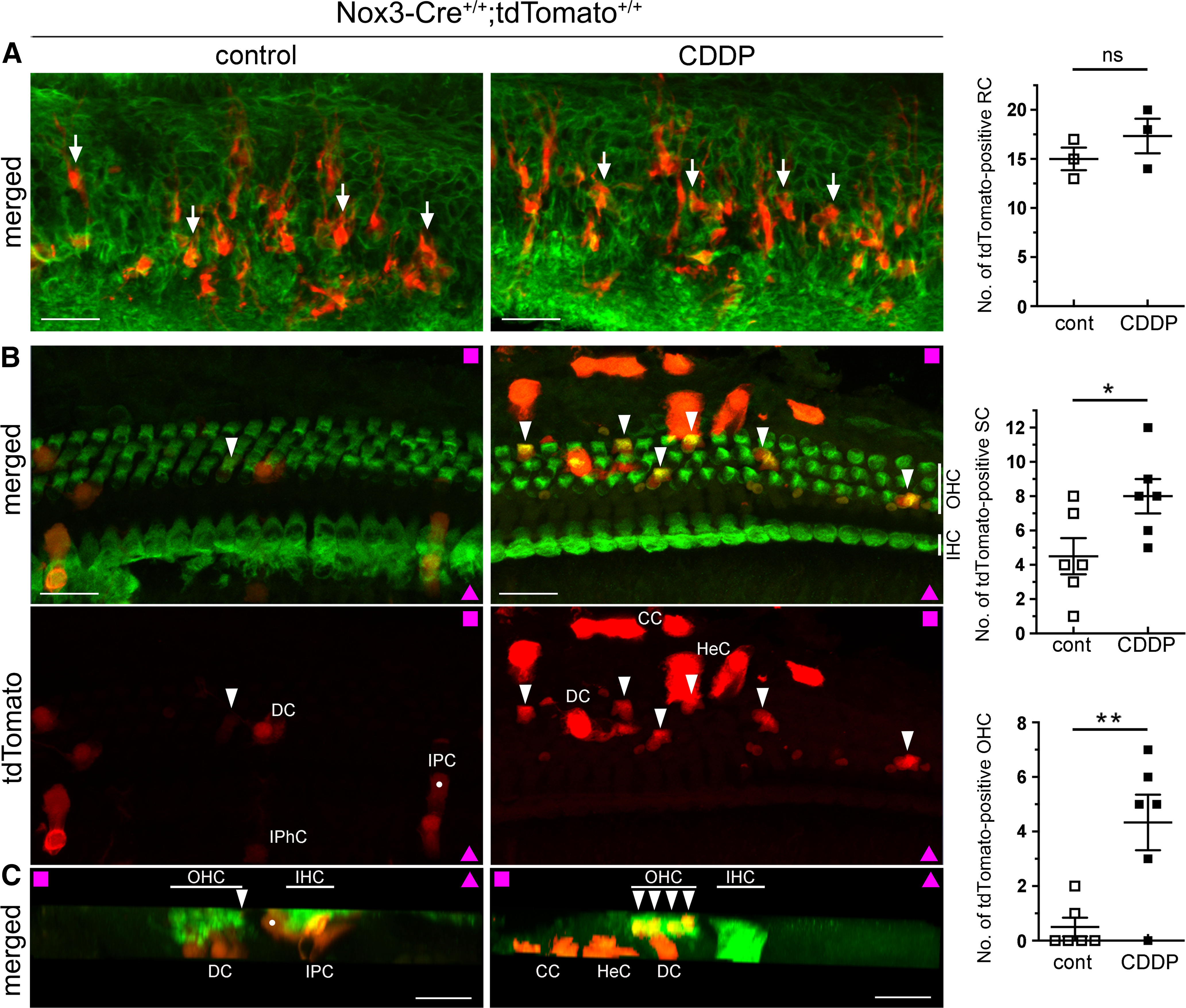
Cisplatin (CDDP)-induced Nox3 expression in OHCs and SCs is accompanied with OHC loss. Surface preparations of the lateral wall (***A***) and the OC at the basal turn (510°, ***B***) from 2-month-old *Nox3-Cre*^+/+^*;tdTomato*^+/+^ (*Nox3*-KO) mice after treatment with NaCl (control) or CDDP stained with Alexa-488-labeled phalloidin (***A***, green) and myosin-VIIa (***B***, green), respectively. After images of ***B*** (stacked axial images in *xy* axis) were obtained, images of ***C*** (stacked sagittal images in *yz* axis) were reconstructed. Arrows and arrowheads indicate tdTomato-positive RCs (***A***) and OHCs (***B***), respectively. Magenta rectangles and triangles represent the side of lateral wall and modiolus, respectively. Scale bars: ***A***, 50 μm; ***B***, ***C***, 20 μm. Graphs represent quantification of the numbers of tdTomato-positive RCs, SCs and OHCs, respectively (*n* = 3; top; *p* = 0.3305, *n* = 6; middle; *p = 0.0370, bottom; **p = 0.0052, by Student's *t* test). ns, not significant.

To elucidate why tdTomato expression was not observed in *Nox3-Cre*^+/−^*;tdTomato*^+/+^ OHCs with Nox3-derived ROS production capabilities, we compared them with tdTomato-positive cells at the basal turn (510°) in *Nox3-Cre*^+/+^*;tdTomato*^+/+^ (*Nox3*-KO) mice with no Nox3-derived ROS production. Although the number of tdTomato-positive RCs was not significantly increased on CDDP treatment in 2-month-old *Nox3*-KO mice (*p* = 0.3305, *t*_(4)_ = 1.107; Fig. 7*A*), the number of tdTomato-positive SCs and OHCs significantly increased compared with that in the untreated controls (SCs: *p* = 0.0370, *t*_(10)_ = 2.406; OHCs: *p* = 0.0052, *t*_(10)_ = 3.557; Fig. 7*B*). In sharp contrast to that in *Nox3-Cre*^+/−^*;tdTomato*^+/+^ mice (Fig. 6), tdTomato-positive OHCs were observed in *Nox3*-KO mice even without CDDP treatment (Fig. 7*B*). These results suggest that OHCs with Nox3-driven ROS production potentially induce apoptosis with or without CDDP treatment.

### Link between Nox3 expression and hearing loss induction

**Figure 8.**
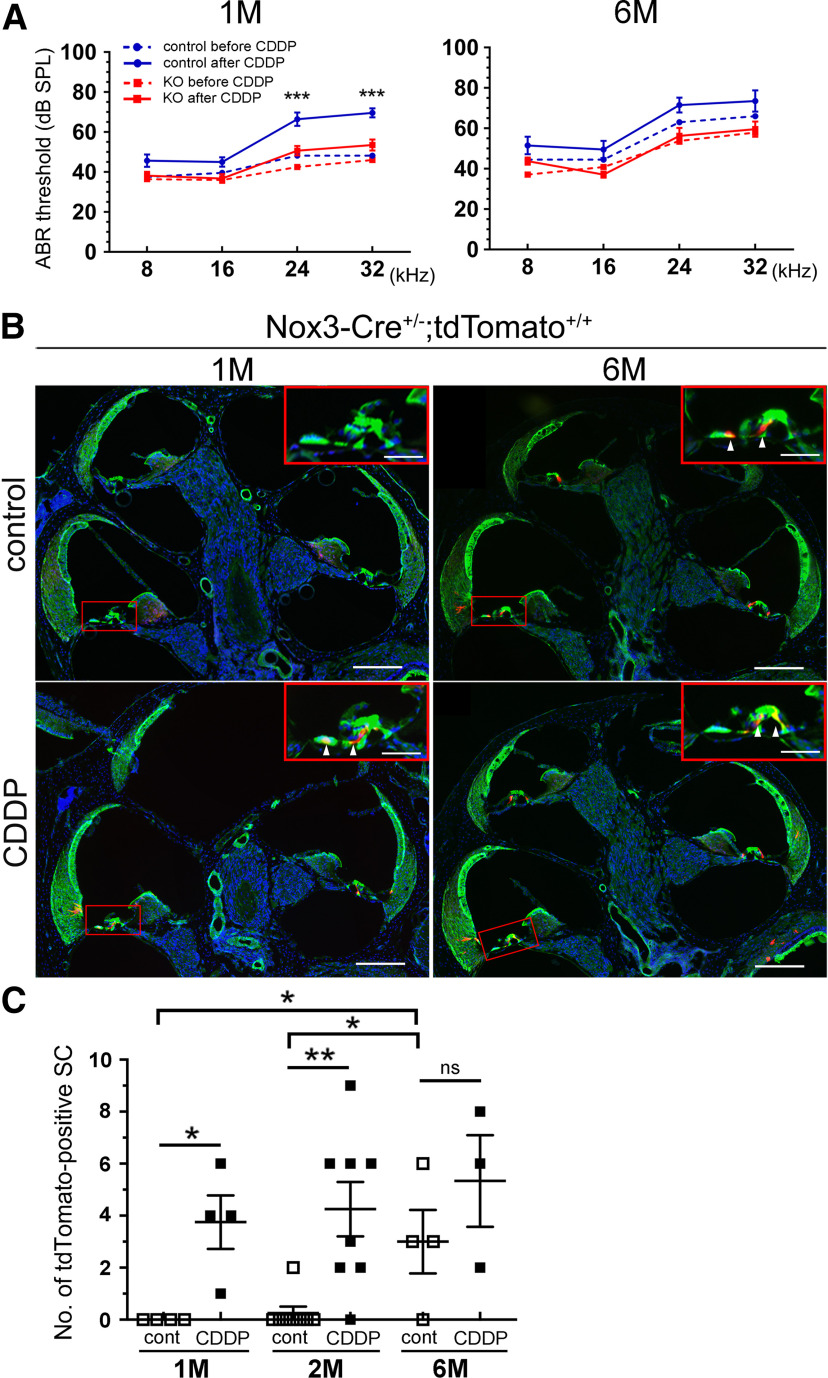
Link between age-related Nox3 expression and cisplatin (CDDP)-induction with hearing loss. ***A***, Pure tone-burst (8, 16, 24, and 32 kHz) ABR thresholds (dB SPL, mean ± SEM) in 1-month-old (M, left, *n* = 7 in control and *Nox3*-KO) and 6 months (right, *n* = 5 and *n* = 6 in control and *Nox3*-KO mice, respectively) mice before and after CDDP treatment. ***p = 0.0009 (24 kHz after CDDP), ***p = 0.0006 (32 kHz after CDDP) in 1 month (control vs KO) by two-way ANOVA with Turkey's *post hoc* test. ***B***, Images of cochlear cryostat sections from 1 month and 6 months *Nox3-Cre*^+/−^*;tdTomato*^+/+^ mice after NaCl (control) or CDDP treatment. Sections were stained with Alexa-488-labeled phalloidin (green) and DAPI (blue). Scale bars, 200 μm. Insets, Magnified images of areas shown by rectangles. Scale bars, 50 μm. Arrowheads indicate tdTomato-positive SCs (red). ***C***, Quantification of the numbers of tdTomato-positive SCs before and after CDDP treatment. After CDDP, there was a significant increase in tdTomato expression in 1 month (*n* = 4, *p = 0.0109) and 2 months (*n* = 8, **p = 0.0023), but not in 6 months (*n* = 4 and *n* = 3 in control and CDDP, respectively; *p* = 0.3105) mice as analyzed by Student's *t* test, compared with the control. Without CDDP treatment, tdTomato-positive SCs significantly increased between 1 month and 6 months, and 2 months and 6 months, respectively (*p = 0.0148 and *p = 0.0103 by one-way ANOVA with Turkey's *post hoc* test). ns, not significant.

We further examined the link between aging and Nox3 induction using CDDP treatment in juvenile (1-month-old) and aged (6-month-old) mice. Similar to that in 2-month-old mice (Fig. 5*B*), CDDP treatment of 1-month-old mice induced significant differences in ABR thresholds at 24 and 32 kHz between control and *Nox3*-KO mice (24 kHz, *p* = 0.0009, *F*_(1,104)_ = 6.132 and 32 kHz, *p* = 0.0006, *F*_(1,104)_ = 6.271; Fig. 8*A*). In contrast, CDDP treatment of 6-month-old mice showed no significant difference between control and *Nox3*-KO mice (Fig. 8*A*). Cryostat sections from 1- and 2-month-old *Nox3-Cre*^+/−^*;tdTomato*^+/+^ mice demonstrated that the number of tdTomato-positive SCs significantly increased after CDDP treatment (1 month, *p* = 0.0109, *t*_(6)_ = 3.638 and 2 months, *p* = 0.0023, *t*_(14)_ = 3.713; Figs. 6*B*, 8*B*,*C*; Table 1). However, the number of tdTomato-positive SCs did not significantly increase after CDDP treatment in 6-month-old *Nox3-Cre*^+/−^*;tdTomato*^+/+^ mice (6 months, *p* = 0.3105, *t*_(5)_ = 1.128; Fig. 8*B*,*C*; Table 1). Consistent with the data from OC surface preparations (Fig. 3*A*,*B*), tdTomato-positive SCs in basal conditions (without CDDP treatment) significantly increased between 1 month and 6 months (0.00 ± 0.00 cells vs 3.00 ± 1.23 cells, *p* = 0.0148, *F*_(2,13)_ = 4.666) and between 2 month and 6 months (0.25 ± 0.25 cells vs 3.00 ± 1.23 cells, *p* = 0.0103, *F*_(2,13)_ = 4.938; Fig. 8*B*,*C*; Table 1). Together, basal Nox3 expression (without treatment/insult) is a critical determinant of ARHL, and aging (6-month-old) reduces CDDP-induced Nox3 expression.

### Nox3 induction at the basal turn of the OC is associated with NIHL

**Figure 9.**
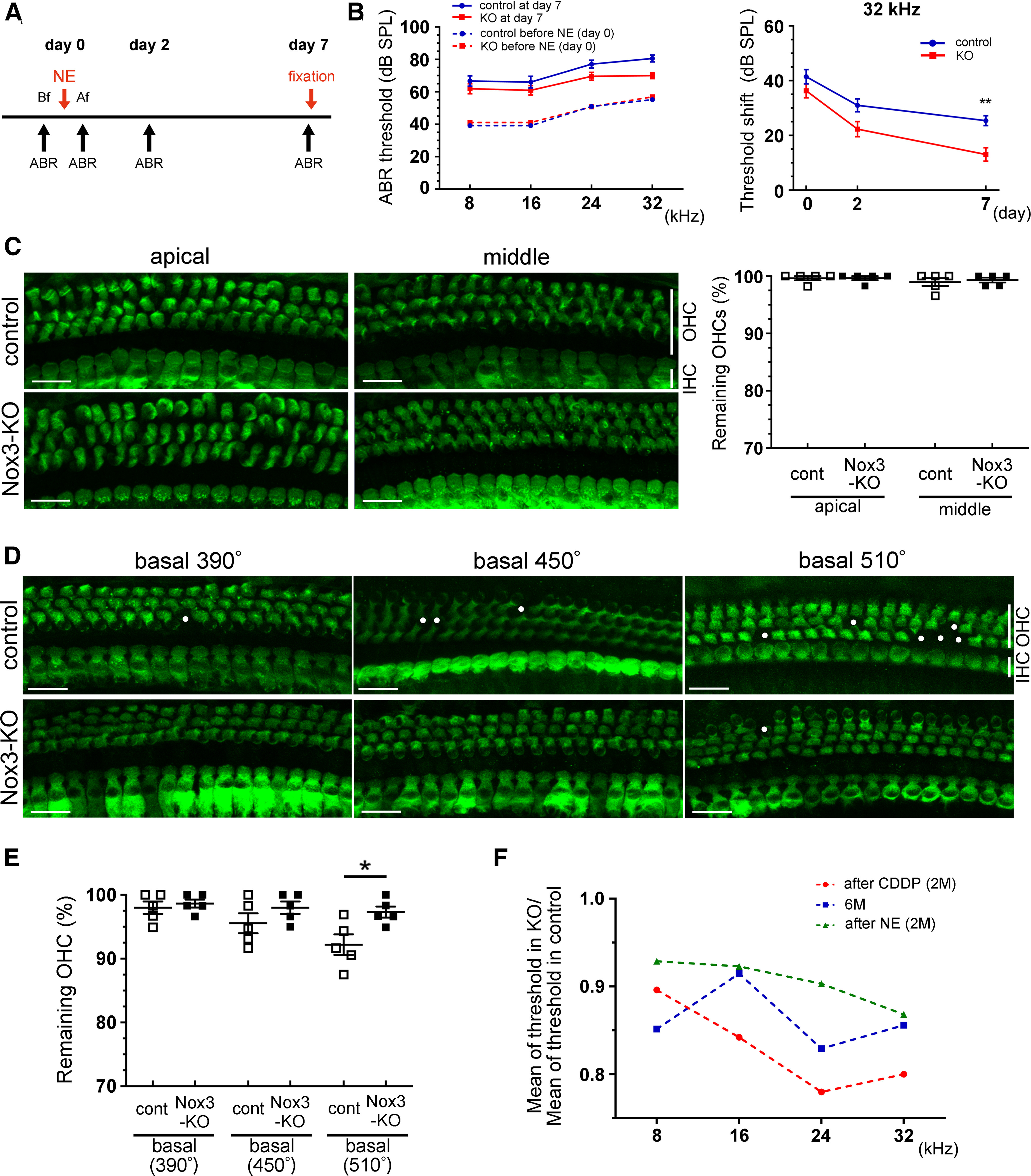
Ameliorated NE-induced hearing loss in *Nox3*-KO mice. ***A***, Chart of ABR measurements and NE (120 dB SPL for 3 h) in 2-month-old (M) control and *Nox3*-KO mice. Bf and Af, immediately before and after NE, respectively. ***B***, Pure tone-burst (8, 16, 24, and 32 kHz) ABR thresholds (dB SPL, mean ± SEM) in control and *Nox3*-KO mice before (day 0) and after NE (day 7). No significant difference was observed between control (*n* = 12) and *Nox3*-KO (*n* = 13) mice (left). ABR threshold shifts (dB SPL, mean ± SEM) were calculated at 32 kHz on days 0, 2, and 7. A significant difference was observed at 32 kHz at day 7 (right; **p = 0.0072) by two-way ANOVA with Turkey's *post hoc* test. ***C–E***, Surface preparations of the OC at the apical and middle (***C***) and basal turn (390°, 450°, and 510°) (***D***) from control and *Nox3*-KO mice at day 7 were stained for myosin-VIIa (green). OHC loss (circles) was detected using confocal microscopy, and the percentages of remaining OHCs were quantified (***C, E***). *n* = 5 and *p = 0.0235 by Student's *t* test. Scale bars, 20 μm. ***F***, The ratio of the mean ABR threshold in *Nox3*-KO mice to the mean ABR threshold in control mice after CDDP (red) or NE (green) treatment with 8, 16, 24, and 32 kHz at 2 months; the ratio of the mean ABR threshold in *Nox3*-KO mice to the mean ABR threshold in control mice (blue) with 8, 16, 24, and 32 kHz at 6 months. The ratio after CDDP treatment was lowest in three groups at 16, 24, and 32 kHz.

To examine whether Nox3 is protective or harmful in NIHL, we exposed 2-month-old control and *Nox3*-KO mice to noise at an intensity of 120 dB SPL for 3 h (Fig. 9*A*). After ABR threshold analysis (Fig. 9*B*, left), we assessed ABR threshold shifts at days 0, 2, and 7 (Fig. 9*B*, right). At day 7, the ABR threshold shift at 32 kHz was significantly lower in *Nox3*-KO mice than in controls (*p* = 0.0072, *F*_(1,144)_ = 4.998; Fig. 9*B*, right). Morphologically, control mice exhibited OHC loss at the basal turn with apical-to-basal progression, which was not observed at the apical and middle turns (Fig. 9*C*,*D*). In contrast, OHC loss in *Nox3*-KO mice was significantly lower at the basal turn (510°) than that measured in control mice (*p* = 0.0235, *t*_(8)_ = 2.793; Fig. 9*D*,*E*). The percentages of remaining OHCs at 510° in control and *Nox3*-KO mice were 92.2 ± 1.6% vs 97.3 ± 0.9% (Fig. 9*E*).

From these results, we show that Nox3-derived ROS play important roles in ARHL, CIHL, and NIHL, especially at high-frequency sounds (Figs. 3*C*, 5*B*, 9*B*). To elucidate how extensively Nox3 is involved in SNHL, we calculated the ratio of the mean ABR thresholds at 8, 16, 24, and 32 kHz. In cases of CIHL and NIHL, the ratio of the mean ABR threshold in *Nox3*-KO mice to the mean ABR threshold in control mice was calculated at 2 months, whereas for ARHL, this ratio was calculated at 6 months (Fig. 9*F*). In this analysis, a lower ratio indicates more involvement of Nox3 in the HL condition. These data suggest that CIHL is most profoundly influenced by Nox3-derived ROS at 16, 24, and 32 kHz, with less influence in ARHL and the least influence in NIHL.

**Figure 10.**
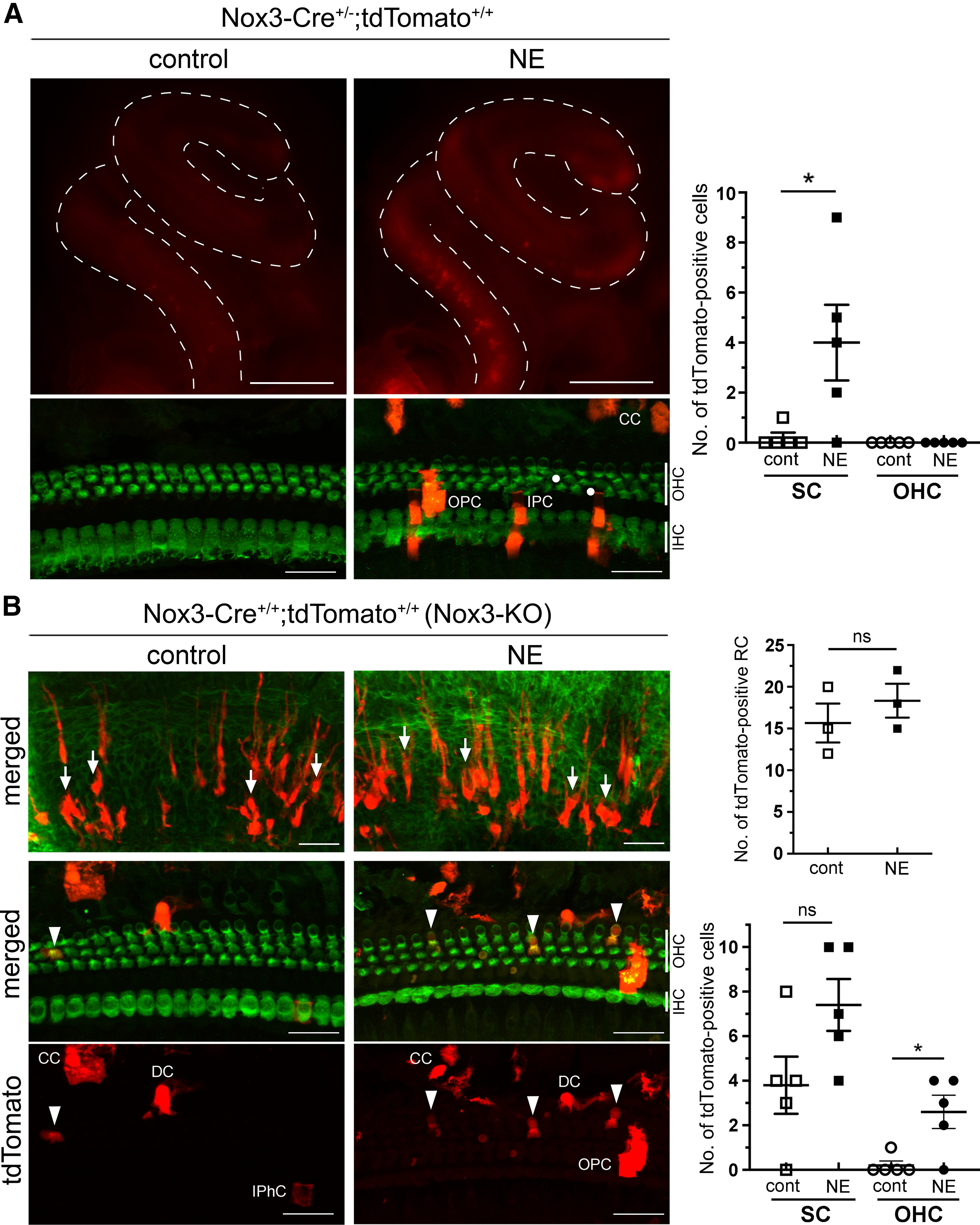
NE-induced Nox3 expression in OHCs accompanied with OHC loss. ***A***, Inner ears of 2-month-old control and noise-exposed *Nox3-Cre*^+/−^*;tdTomato*^+/+^ mice were fixed at day 7. Whole-mount fluorescence images (top panels) showing induced tdTomato expression (red) at the basal turn of the cochlea by NE. Surface preparations at the basal turn (510°, bottom panels) were stained for myosin-VIIa (green). Circles represent OHC loss. Scale bars: top panels, 500 μm; bottom panels, 20 μm. A graph of the quantification of the number of tdTomato-positive SCs and OHCs in the surface preparations at the basal turn (510°) (*n* = 5; *p = 0.0379 by Student's *t* test). ***B***, Inner ears of 2-month-old control or noise-exposed *Nox3-Cre*^+/+^*;tdTomato*^+/+^ (*Nox3*-KO) mice were fixed at day 7. Surface preparations at the basal turn (510°) were stained for phalloidin (top panels, green) and myosin-VIIa (middle and bottom panels, green), respectively. Arrows indicate tdTomato-positive RCs (red). Arrowheads indicate tdTomato-positive OHCs. Scale bars: top panels, 50 μm; middle and bottom panels, 20 μm. Quantification of the number of tdTomato-positive RCs, SCs, and OHCs (top: *p* = 0.4370; *n* = 3, bottom: *p* = 0.0713 in SCs and *p = 0.0147 in OHCs; *n* = 5, by Student's *t* test). ns, not significant

Next, we examined how Nox3 expression is affected by NE using 2-month-old *Nox3-Cre*^+/−^*;tdTomato*^+/+^ mice. Similar to that with CDDP treatment, NE induced Nox3 expression predominantly at the basal turn of the cochlea (Fig. 10*A*). In surface preparations of the OC at the basal turn (510°), there was a significant increase in tdTomato-positive SCs, but not OHCs, which was accompanied by OHC loss (*p* = 0.0379, *t*_(8)_ = 2.484; Fig. 10*A*). To further examine Nox3 expression in OHCs, 2-month-old *Nox3-Cre*^+/+^*;tdTomato*^+/+^ (*Nox3*-KO) mice were exposed to NE. Although the number of tdTomato-positive RCs and SCs was not significantly increased by NE (RC: *p* = 0.4370, *t*_(4)_ = 0.8627; SC: *p* = 0.0713, *t*_(8)_ = 2.078; Fig. 10*B*), the number of tdTomato-positive OHCs was significantly increased by NE (*p* = 0.0147, *t*_(8)_ = 3.098; Fig. 10*B*). Similar to CIHL, these results suggest that OHCs with Nox3-driven ROS production are susceptible to cell death after NE. Additionally, the NE-induced Nox3 expression at the basal turn in the OC is one of the factors causing NIHL.

## Discussion

Previously, p22*^phox^* was reported to be expressed from E14 to P12, indicating that otoconia are formed during this period (Nakano et al., 2008). p22*^phox^* is also expressed at the ES and ED, which are not sites of otoconia biosynthesis (Nakano et al., 2008). In this study, we used *Nox3-Cre;tdTomato* mice to directly show that Nox3-derived ROS are primarily generated from nonsensory epithelia facing the lumens of the ES and ED (Paffenholz et al., 2004). Additionally, we found that Nox3 is expressed in nonsensory epithelia of semicircular canals and vestibules facing the lumens. Interestingly, not all epithelia of the ES, ED, semicircular canals, or vestibules expressed Nox3. Therefore, the mechanism governing otoconia formation in response to Nox3-derived ROS remains unclear. Pendrin is a HCO_3_^−^/Cl^−^ exchanger whose expression is similar to that of Nox3 in the ES, ED, RCs, transitional nonsensory epithelia in utricles, saccules, and ampullae, but not the SV (Everett et al., 1999). Its deletion causes otoconia defects and occasional giant otoconia (Everett et al., 2001). The exact effects of reduced Nox3 expression in semicircular canals and vestibules other than the ES and ED are unknown; however, one possibility is that it functions to maintain appropriate ROS concentration gradients between the ES/ED and vestibule. Indeed, ectopic otoconia in embryonic rodents (Ignatova et al., 2004) and adult birds (Malkemper et al., 2018) have been reported. These phenomena might be caused by the abnormal longitudinal flow of otoconin-90 (Lundberg et al., 2015) from the vestibule to the ES (Ignatova et al., 2004), suggesting that otoconia formation occurs at regions with specific conditions.

We discovered that Nox3 is also expressed in RCs during cochlea development (Figs. 1*D*, 2*A*) without significant CDDP- or NE-dependent responses. RCs reportedly regulate the ion composition of the endolymph and safeguard cochlear homeostasis (Jagger and Forge, 2013). Pendrin has been reported to be expressed in RCs (Choi et al., 2011), suggesting that Nox3 in RCs might also be involved in otoconia formation. Thus, Nox3 in RCs does not significantly affect SNHL, at least not CIHL and NIHL. However, the exact function of Nox3 in RCs requires further study.

**Figure 11.**
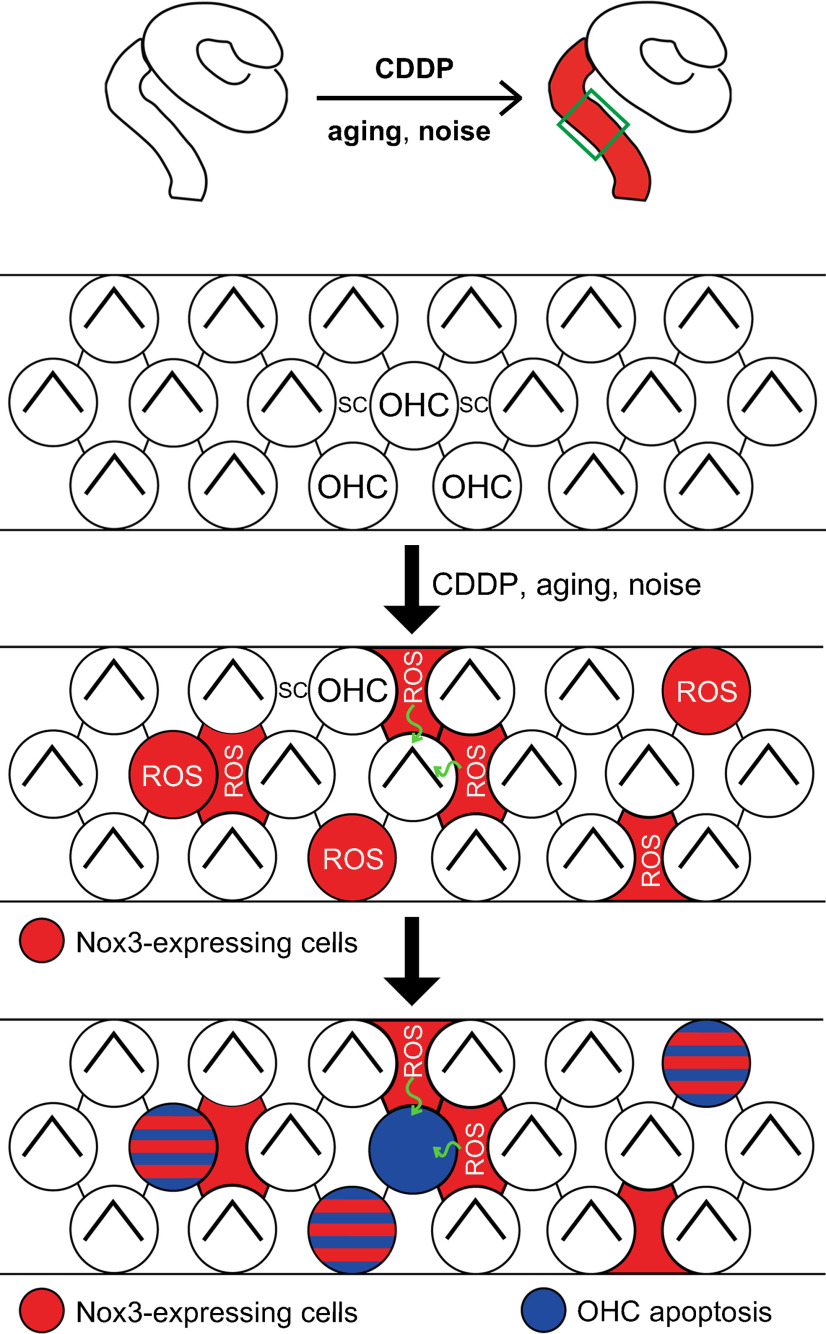
Proposed model of OHC loss caused by the extracellularly released Nox3-derived ROS from OHCs and SCs. Illustration represents the mechanism of OHC loss by Nox3-derived ROS, which was dominantly induced at the basal turn of the cochlea by cisplatin (CDDP), aging, and noise. In the OC, CDDP, aging, or noise increased Nox3 in OHCs and surrounding SCs; additionally, Nox3-derived ROS was released from and into OHCs and SCs. OHCs, but not SCs, showed ROS-induced apoptosis from endogenously produced ROS and/or that of surrounding SCs. Nox3-derived ROS produced by SGNs contributes to their apoptosis at the SG (Fig. 5*E*,*F*).

Several acquired forms of HL have previously been associated with Nox-generated ROS (Wong and Ryan, 2015). In mice cochleae, only *Nox3* mRNA was detected in baseline conditions by RT-PCR, whereas *Nox1*, *Nox4*, and *Duox2* mRNA were detectable after CDDP treatment, although *Duox2* expression was still low compared with the other genes (Kim et al., 2010). CDDP-induced apoptosis in the OC and SGNs was found to be inhibited by the TNFα neutralizer etanercept (Kim et al., 2010); however, that study did not elucidate which Nox isoform (Nox1, Nox3, or Nox4) was the main inhibitory target of etanercept. Studies using a rat model indicate the presence of a Nox3-associated signaling axis, in which Nox3 activation is coupled with the upregulation of transient receptor potential vanilloid 1 (TRPV1) and signal transducer and activator of transcription-1 (STAT1), resulting in TNFα-mediated inflammation and apoptosis in CIHL (Mukherjea et al., 2010; Kaur et al., 2016). Transtympanic administration of *TRPV1* (Mukherjea et al., 2008), *Nox3* (Mukherjea et al., 2010), or *STAT1* siRNA (Mukherjea et al., 2011) offers protection from CIHL. The Nox3-dependent NIHL pathway is involved in the synergistic activation of Nox3 and TRPV1 and TNFα-associated inflammation, which results in cell damage (Dhukhwa et al., 2019). In rat cochleae, Nox2 protein was detectable after neomycin treatment and contributed to OHC vulnerability in the third row (Qi et al., 2018). Nox3 expression, detected using a Nox3 antibody, reportedly increased in the OC, SG, and SV in rats with D-galactose-induced aging (Du et al., 2012). More recently, *Nox3* expression in the SG and SV in mice was reported using RNAscope (Rousset et al., 2020). The same group showed that *p22^phox^*-deficient mice are resistant to age-related ribbon synaptic loss and HL (Rousset et al., 2020). Thus, involvement of Nox1, Nox2, Nox3, and Nox4 has been reported in SNHL; however, details such as the degree of involvement of each Nox isoform in each SNHL type remain unclear. Herein, we demonstrate the presence of Nox3-expressing cells in cochleae and confirm that Nox3 expression increases with ARHL, CIHL, and NIHL in the basal turns of cochleae, particularly in SCs (Table 1). Furthermore, tdTomato-positive OHCs were detected in *Nox3-Cre*^+/+^*;tdTomato*^+/+^, but not in *Nox3-Cre*^+/−^*;tdTomato*^+/+^, mice before and after CDDP treatment, suggesting that OHCs with Nox3-derived ROS are susceptible to apoptosis even without CDDP treatment. Nox3 is expressed on the plasma membrane and induces the release of extracellular superoxide in overexpressing cell models (Ueyama et al., 2006); here, we illustrate the mechanism by which Nox3-derived ROS from OHCs and SCs are involved in OHC loss (Fig. 11). However, the auditory neuropathy mechanism induced by Nox3 might be involved in ARHL to some extent (Rousset et al., 2020) because tdTomato-positive SGNs were detected and found to increase with aging (Table 1).

Regarding the mechanism of ARHL, CIHL, and NIHL in association with Nox3-derived ROS, a conserved number of ribbon synapses was reported in 26-week-old *p22^phox^*-deficient mice compared with that in controls (Rousset et al., 2020). We also observed preserved numbers of ribbon synapses and OHCs in basal turns in 6-month-old *Nox3*-KO mice compared with those in controls. Although both human and mouse Nox3-p22*^phox^* heterodimers are constitutively active without stimulation (i.e., Nox3 activity is likely regulated by expression of Nox3-p22*^phox^* and/or its activator proteins) (Leto et al., 2009), their activation mechanisms vary. Activation of human NOX3 requires NOXO1 alone, whereas mouse Nox3 requires Noxo1 and Noxa1 (Banfi et al., 2004; Ueyama et al., 2006). Moreover, physiological functions of Nox3 in cochleae are still unknown as *Nox3*-KO mice showed normal morphologic and functional cochlea development. Nox3 activation, its physiological roles in cochleae, the mechanism by which Nox3-derived ROS decrease ribbon synapse numbers, and the association of these factors with HL and HC loss (Kujawa and Liberman, 2019; Jeng et al., 2020) must be addressed to develop therapeutics for Nox3-related SNHL.

Using *Nox3*-KO mice, we showed that Nox3 is significantly involved in CIHL, and to a lesser degree, ARHL and NIHL. CIHL is more severe in children younger than 5 years than in children older than 15 years (Li et al., 2004). Similarly, age-dependent sensitivity was observed in our mouse model. Notably, age-related CIHL can be explained by age-related Nox3 expression in cochleae and its higher and lower induction by CDDP in young and aged mice, respectively. Additionally, aged mice might acquire protective mechanisms against CDDP during age-related Nox3 induction. The present study and others (Dhukhwa et al., 2019) show that Nox3 has a deteriorative effect during NIHL, especially at high-frequency sounds, although a previous report showed a mildly protective effect of Nox3 in NIHL at low-frequency sounds (Lavinsky et al., 2015). A possible explanation for this discrepancy is the NE conditions used, but we found that Nox3 involvement in NIHL is weaker than that in CIHL and ARHL.

In our reporter mice, at least one *Nox3* allele was replaced by *Cre*. We cannot exclude the effect of Cre in this system and the possibility that it alters Nox3 expression patterns (among *Nox3-Cre*^+/+^*;tdTomato*^+/+^, *Nox3-Cre*^+/−^*;tdTomato*^+/+^, and *Nox3-Cre*^−/−^*;tdTomato*^+/+^ mice). Indeed, there are some differences between our findings and those of another group (Rousset et al., 2020), such as strong, weak, and no expression of Nox3 in the SG, SV, and HCs, respectively, in the aforementioned study. Regarding Nox3 expression in SGNs, we detected moderate Nox3 expression (Table 1). Our reporter mice and a report using RT-PCR (Banfi et al., 2004) showed no *Nox3* expression in the SV. We observed no apoptotic cells in the SV with CDDP treatment, a finding consistent with some reports (Wang et al., 2003; van Ruijven et al., 2004) and inconsistent with others (Mukherjea et al., 2010). Wang et al. reported high variability in the extent of damage to the SV in individual guinea pigs (Wang et al., 2003). Regarding the discrepancy in Nox3 expression in HCs, our proposed OHC loss mechanism (Fig. 11) and restricted Nox3 expression in IHCs of aged mice probably explain the lack of Nox3 in WT HCs (Rousset et al., 2020). Some of these differences might be attributed to different rodent or genetic backgrounds. As per DNA microarray data using P4 WT and hetero *Nox3*-KO mice, *Nox3* mRNA levels were not significantly different (321 in WT vs 305 in hetero *Nox3*-KO). Additionally, *p22^phox^* mRNA levels were 374 in WT vs 329 in hetero *Nox3*-KO. Thus, *Nox3-p22^phox^* mRNA expression levels do not vary between these genotypes. Consistent with these results, the “head-tilt” phenotype and otoconia defect are only observed in *Nox3*-KO, but not hetero *Nox3*-KO, mice, showing that Nox3 expression patterns are not significantly different between WT and hetero *Nox3*-KO mice.

In conclusion, we identified Nox3-expressing regions and cell types in inner ears using *Nox3-Cre;tdTomato* mice, which are particularly useful as no reliable Nox3 antibodies are available. We also found that Nox3 upregulation in SCs and OHCs, especially in basal turns, is directly involved in SNHL development. We propose that Nox3 inhibition in cochleae is a promising approach to prevent acquired SNHL.
